# Quinoline based receptor in fluorometric discrimination of carboxylic acids

**DOI:** 10.3762/bjoc.4.52

**Published:** 2008-12-17

**Authors:** Kumaresh Ghosh, Suman Adhikari, Asoke P Chattopadhyay, Purnendu Roy Chowdhury

**Affiliations:** 1 Department of Chemistry, University of Kalyani, Kalyani, Nadia-741235, India; 2 Chembiotek Research International Pvt. Ltd., Salt Lake City, Kolkata-700 091, India

**Keywords:** carboxylic acid recognition, excimer emission, naphthalene, quinoline

## Abstract

Quinoline and naphthalene-based fluororeceptors **1** and **2** have been designed and synthesized for detection of hydroxy carboxylic acids in less polar solvents. The receptor **1** shows monomer emission quenching followed by excimer emission upon hydrogen bond-mediated complexation of carboxylic acids. The excimer emission distinguishes aromatic dicarboxylic acids from aliphatic dicarboxylic acids and even long chain aliphatic dicarboxylic acids from short chain aliphatic dicarboxylic acids. The receptor **1** is found to be selective for citric acid with a strong excimer emission in CHCl_3_. On the contrary, the receptor **2** exhibited less binding constant value and did not form any excimer upon complexation with the same acids under similar conditions. This established the role of quinoline ring nitrogen in binding with the acids.

## Introduction

The sensing and monitoring of ions and molecules by designed synthetic receptors is currently of major interest in the area of molecular recognition [1–3]. Among various sensing techniques available for clinical, biological and environmental analyses, fluorescence sensing is unique because of high sensitivity and compatibility for online and real-time analyses [4]. A large number of examples of fluorescent sensors capable of sensing ions and molecules have appeared over the past few years [5–7]. In this context, fluorescent sensors, which rely on guest-induced folding of flexible receptors bringing the fluorophore probes close enough to function as an excimer, are proved to be useful to read out the molecular recognition process more conveniently [8–9].

**Figure 1:**
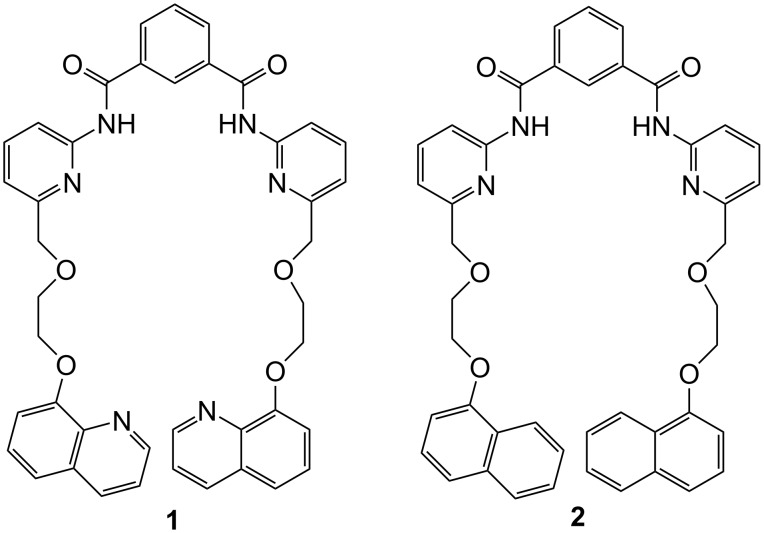
Structures of compounds **1** and **2**.

The recognition and sensing of carboxylic acids has attracted considerable attention owing to their important role in biology [10]. The recognition of both mono- and dicarboxylic acids by a large number of receptors of different architectures is known [11–14]. We have also reported a series of synthetic receptors for carboxylic acids of various types [15–18]. In continuation, we report here a new quinoline based sensor **1** (Figure 1) which is able to bind citric, gluconic and tartaric acids strongly in the less polar solvent CHCl_3_. The guests can be clearly distinguished by observing the strong excimer emission formed by the pendant quinoline probes upon complexation. To establish the role of quinoline ring nitrogen in complexation we also report here an alternative naphthalene-based receptor **2** (Figure 1), where quinoline has been replaced by naphthalene keeping all the other hydrogen bonding groups fixed. The key in all the designs is the appropriate flexible ether linkage to hold the fluorophore probes viz. quinoline and naphthalene in such a manner as to create open clefts of different topologies.

## Results and Discussion

### Synthesis

**Scheme 1:**
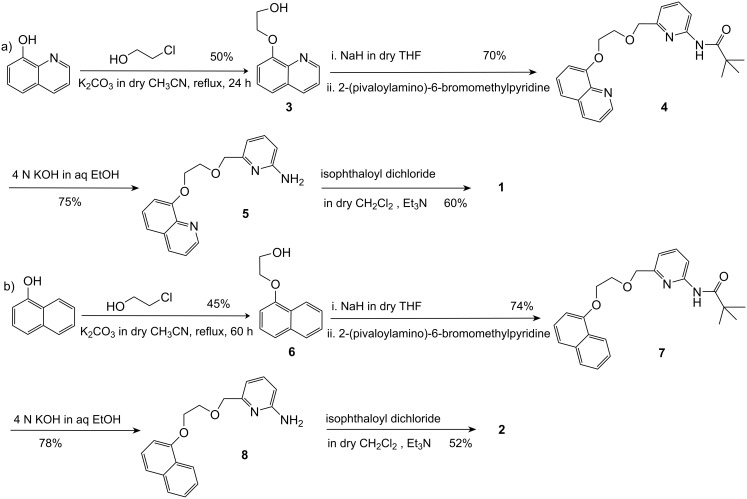
Syntheses of receptors **1** and **2**.

The receptors **1** and **2** were synthesised according to Scheme 1. The alcohols **3** and **6**, obtained from 8-hydroxyquinoline and 1-naphthol, were coupled with 2-(pivaloylamino)-6-bromomethylpyridine [obtained from 2-(pivaloylamino)-6-methylpyridine by reaction with NBS in dry CCl_4_] to give compounds **4** and **7**, respectively. Amide hydrolysis of **4** and **7** afforded the corresponding amines **5** and **8** in good yields. Coupling of these amines with isophthaloyl dichloride yielded the desired receptors **1** and **2**. All the compounds were characterized using ^1^H NMR, ^13^C, mass, IR and UV spectroscopic methods.

### Interaction studies

#### UV-vis study

**Figure 2:**
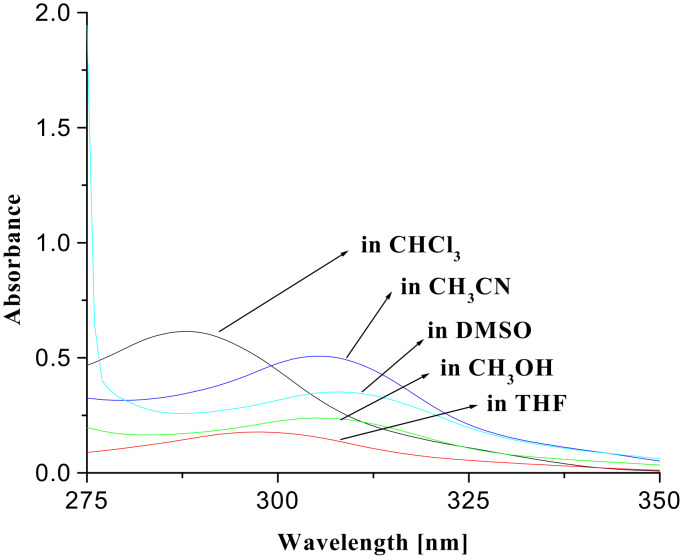
UV-vis spectra of **1** (*c* = 5.05 × 10^−5^ M) in different solvents.

**Figure 3:**
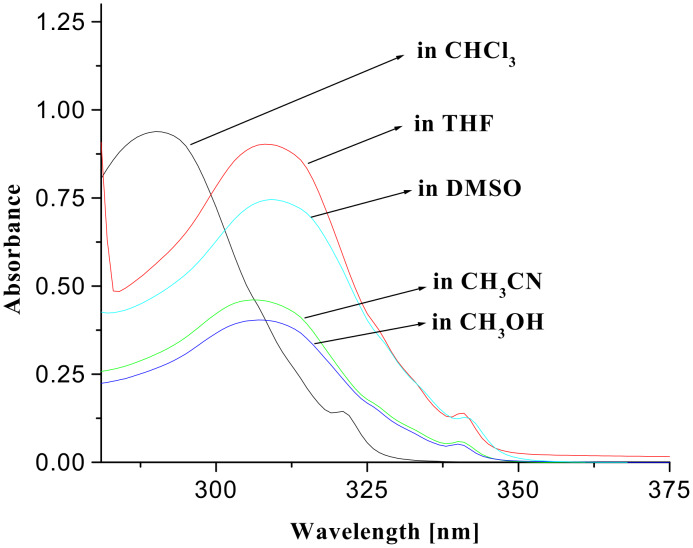
UV-vis spectra of **2** (*c* = 5.05 × 10^−5^ M) in different solvents.

The sensitivity and selectivity of the receptors **1** and **2** were evaluated by observing the changes in ^1^H NMR, UV-vis and fluorescence emission in CHCl_3_. Initially, the photophysical behaviors of the receptors **1** and **2** were noticed in solvents of different polarities. In the ground state the absorption peak at 289 nm for quinoline of **1** and at 290 nm for naphthalene of **2** in CHCl_3_ are considerably affected in intensities as well as positions (red shift; ~18 nm) as the solvent polarity is varied (see Figure 2 and Figure 3).

**Figure 4:**
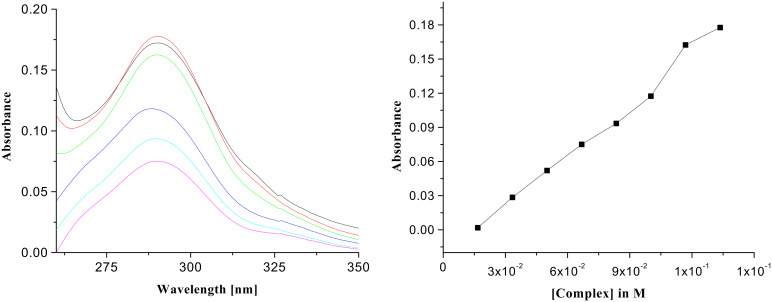
UV spectra of the complex of **1** with citric acid (*c* = 1.67 × 10^−5^ M) and its change of absorbance on dilution (left side); plot of absorbance vs. concentration of the complex of citric acid with **1** (right side).

**Figure 5:**
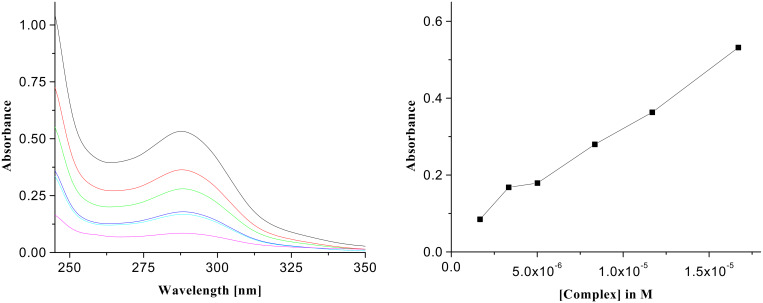
UV spectra of the complex of **1** with D-(−)-tartaric acid (*c* = 1.67 × 10^−5^ M) and its change of absorbance on dilution (left side); plot of absorbance vs. concentration of the complex of D-(−)-tartaric acid with **1** (right side).

The absorption spectra of **1** and its 1:1 complexes with citric, D-(−)-tartaric, D-(−)-gluconic, succinic, glutaric and terephthalic acids in CHCl_3_ were recorded to investigate the interactions in the ground state. Chloroform solutions of the 1:1 complexes were diluted gradually with chloroform and the change in intensity, as a function of the complex concentration, was linear in each case. Figure 4, for example, shows the effect of dilution on the UV spectra of the 1:1 complex of citric acid with **1**. In the 1:1 complex of **1**, the absorption at 290 nm is significantly reduced. The change in absorbance with complex concentration is found to be linear (Figure 4, right side). Figure 5 indicates the case of **1** with D-(−)-tartaric acid where a similar nature of interaction is attributed. These changes in the UV-vis spectra were used conveniently to study the binding since lower concentration led to a more accurate determination of the values of the association constants for the acids (Table 1) [19]. Citric acid, a tricarboxylic acid with more hydrogen bonding groups, shows a higher binding constant than D-(−)-gluconic and D-(−)-tartaric acids. D-(−)-Gluconic acid with more -OH groups in the backbone, exhibits a value of 1.55 × 10^5^ M^−1^ which is slightly less than tartaric and citric acids. The non-hydroxy dicarboxylic acids such as succinic, glutaric, terephthalic acids bind weakly compared to the hydroxy acids. Besides the dilution method we also followed a continuous variation method where the absorbance of receptor **1** was monitored as a function of guest concentration [20]. In order to do so the receptor was dissolved in CHCl_3_ and the carboxylic acid guest, dissolved in CHCl_3_ containing 0.8% DMSO, was gradually added to the receptor solution. The corresponding change in absorption of the receptor was noted after each addition. The binding constant values determined by this method are found to be less (Table 1) due to the presence of DMSO, a competitive hydrogen-bonding partner which reduces the binding affinity.

**Table 1:** Association constants of **1** by UV method.

Guest acid	*K*_a_ [M^−1^]^a^	*K*_a_ [M^−1^]^b^

Citric acid	3.01 × 10^5^	2.98 × 10^4^
D-(−)-Tartaric acid	2.78 × 10^5^	2.12 × 10^4^
D-(−)-Gluconic acid	1.55 × 10^5^	1.67 × 10^4^
Succinic acid	4.45 × 10^4^	3.29 × 10^3^
Glutaric acid	7.34 × 10^4^	5.04 × 10^3^
Adipic acid	7.26 × 10^4^	9.23 × 10^3^
Sebacic acid	2.98 × 10^3^	1.96 × 10^3^
Terephthalic acid	2.00 × 10^4^	4.33 × 10^3^

^a^Determined in dry CHCl_3_ by dilution method (at wavelength 290 nm). ^b^Determined in dry CHCl_3_ by adding guests dissolved in CHCl_3_ containing 0.8% DMSO (at wavelength 290 nm).

**Figure 6:**
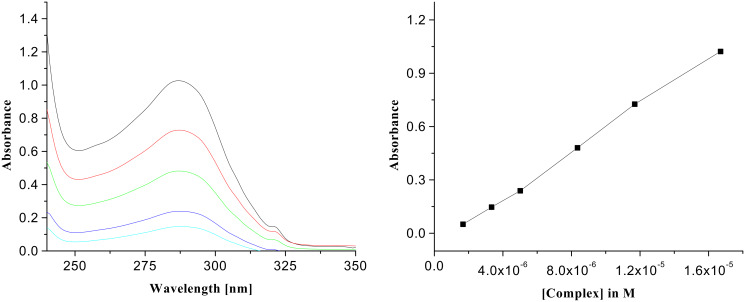
UV spectra of the complex of **2** with citric acid (*c* = 1.67 × 10^−5^ M) and its change of absorbance on dilution (left side); plot of absorbance vs. concentration of the complex of citric acid with **2** (right side).

**Figure 7:**
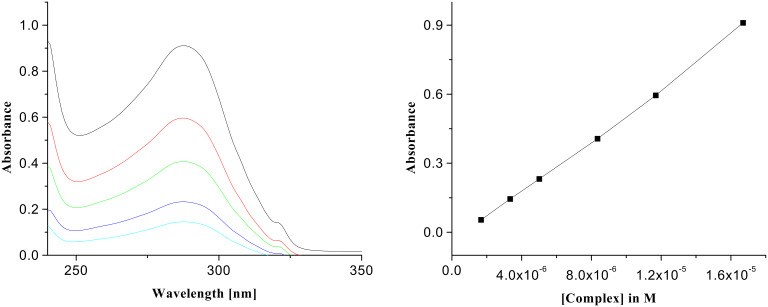
UV spectra of the complex of **2** with D-(−)-tartaric acid (*c* = 1.67 × 10^−5^ M) and its change of absorbance on dilution (left side); plot of absorbance vs. concentration of the complex of D-(−)-tartaric acid with **2** (right side).

Interestingly, as we move from receptor **1** to receptor **2**, the binding constant value is also reduced due to fewer hydrogen bonds being formed during complexation owing to the replacement of quinoline moiety in **1** by naphthalene, which does not take part in complexation. To ascertain the binding potencies, UV titrations of the receptor **2** in presence of the same guests were carried out in CHCl_3_. The change in absorbance of the complexes of **2** with the acid guests on dilution with CHCl_3_ was linear in each case. Figure 6 and Figure 7, for example, demonstrate the changes in absorbance of the 1:1 complexes of citric acid and D-(−)-tartaric acid respectively with receptor **2**. The binding constant values are collected in Table 2.

**Table 2:** Association constants of **2** by UV method.

Guest acid	*K*_a_ [M^−1^]^a^	*K*_a_ [M^−1^]^b^

Citric acid	2.64 × 10^4^	4.86 × 10^3^
D-(−)-Tartaric acid	3.36 × 10^4^	7.70 × 10^3^
D-(−)-Gluconic acid	1.55 × 10^4^	5.28 × 10^3^
Succinic acid	1.41 × 10^4^	7.78 × 10^3^
Glutaric acid	1.66 × 10^4^	1.54 × 10^3^
Adipic acid	1.99 × 10^4^	8.11 × 10^3^
Sebacic acid	1.02 × 10^3^	2.83 × 10^3^
Terephthalic acid	1.09 × 10^4^	7.77 × 10^3^

^a^Determined in dry CHCl_3_ by dilution method (at wavelength 290 nm). ^b^Determined in dry CHCl_3_ by adding guests dissolved in CHCl_3_ containing 0.8% DMSO (at wavelength 290 nm).

#### Fluorescence study

**Figure 8:**
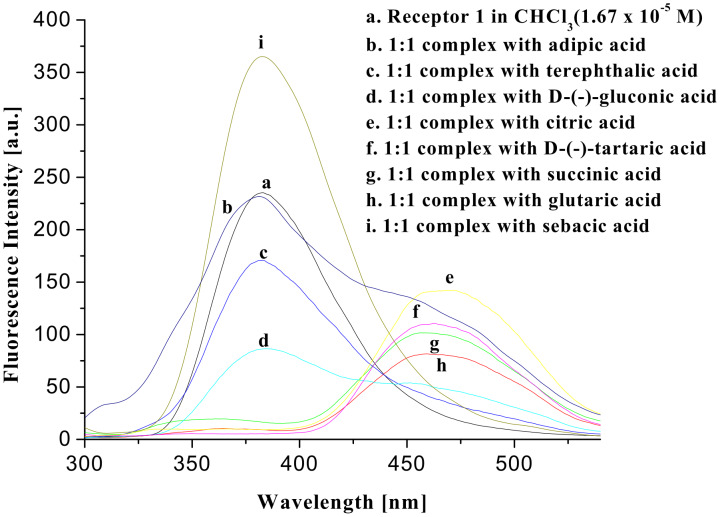
Fluorescence change of **1** in CHCl_3_ in the presence of carboxylic acids (λ_ex_ = 290 nm).

To ascertain their excited state properties, fluorescence spectra of the receptor **1** were recorded in CHCl_3_ both in the presence and absence of the guest acids. Figure 8 shows the fluorescence spectra of receptor **1** and its 1:1 complexes with citric, D-(−)-tartaric, D-(−)-gluconic, succinic, glutaric, adipic, sebacic and terephthalic acids. On complexation, fluorescence quenching of the monomer emission occurs significantly with simultaneous generation of a new peak at longer wavelength, presumably due to formation of excimer. The degree of quenching and the appearance of excimer are dependent on the nature of the acids. The nonhydroxy dicarboxylic acids of different chain lengths are less efficient in forming strong excimers than hydroxy di- and tricarboxylic acids. Terephthalic acid, an example of aromatic dicarboxylic acid, on the contrary, did not produce any excimer upon complexation. This is attributed to the rigidity and steric features of the aromatic diacid that fails to bring the pendant quinolines close enough for excimer formation. We also tested the possibility of excimer formation in the presence of different aliphatic dicarboxylic acids of different chain lengths. It was interesting that the excimer was noticed only in the presence of succinic, glutaric and adipic diacids. In the presence of diacids of higher chain length such as suberic acid the peak for the excimer at higher wavelength did not appear. This observation is thus interesting and useful in distinguishing aliphatic from aromatic dicarboxylic acids and also aliphatic dicarboxylic acids of specific chain lengths.

**Figure 9:**
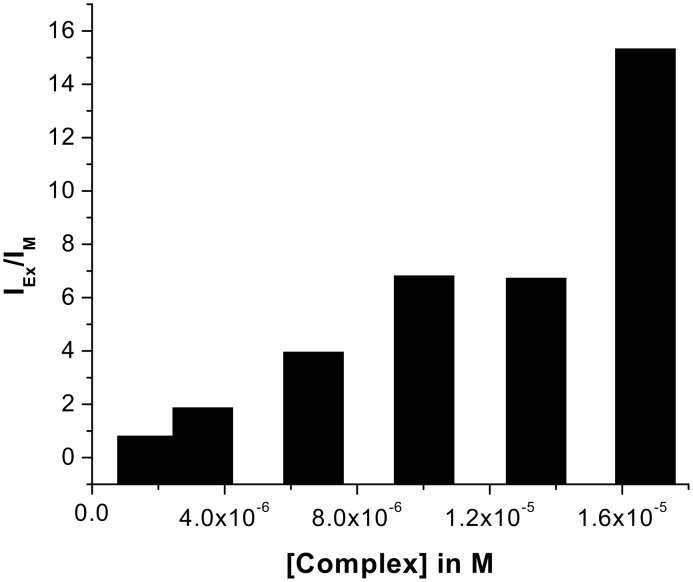
Plot of the ratio of excimer to monomer emission vs concentration of the complex of **1** with citric acid.

**Figure 10:**
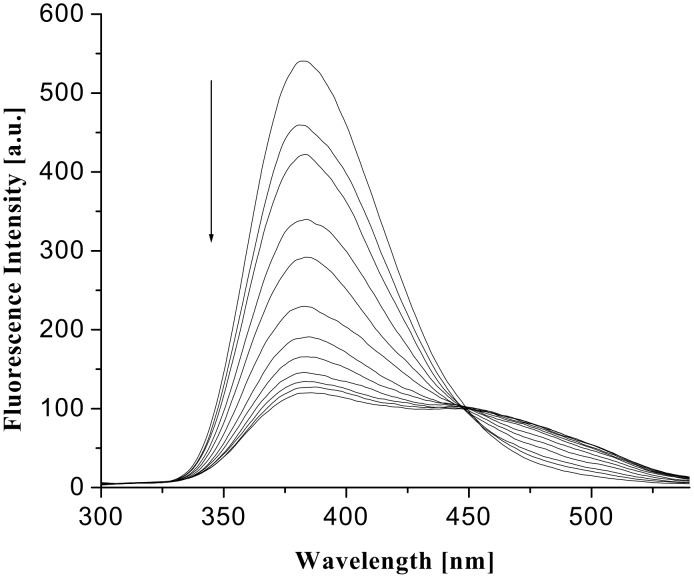
Fluorescence change of **1** in CHCl_3_ (*c* = 1.67 × 10^−5^ M) upon addition of citric acid dissolved in CHCl_3_ containing 0.8% DMSO (λ_ex_ = 290 nm).

**Figure 11:**
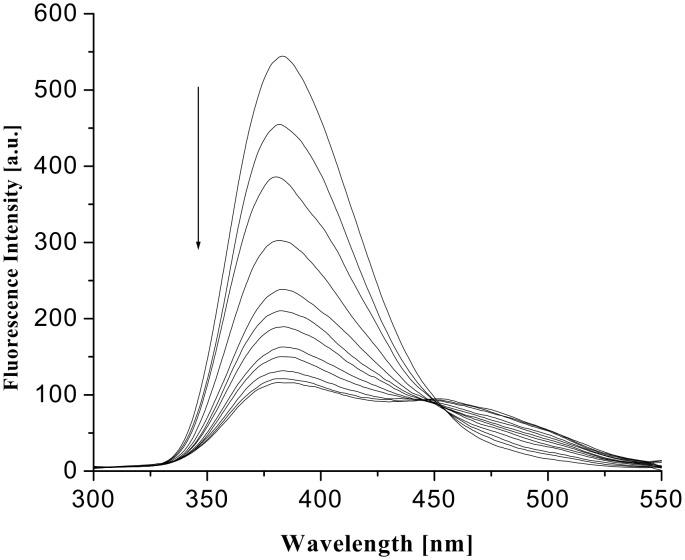
Fluorescence change of **1** in CHCl_3_ (*c* = 1.67 × 10^−5^ M) upon addition of D-(−)-tartaric acid dissolved in CHCl_3_ containing 0.8% DMSO (λ_ex_ = 290 nm).

The excimer emission resulted from the intramolecular excimer, rather than intermolecularly, as indicated by the dilution experiments at different concentrations in which the intensities of the ratio of excimer to monomer emission changed gradually (Figure 9). The formation of strong excimers in the presence of hydroxy dicarboxylic acids could be attributed to the guest acid templated hydrogen bond induced organization of the pendant quinoline moieties that are linked to the pyridine motifs through flexible ether chains. This was further confirmed by a control experiment using propanoic acid. Propanoic acid as a monocarboxylic acid is preferentially complexed into the pyridine amide sites and the lower rim of **1** remains non-interacting giving no excimer emission. It is notable that this excimer formation is dependent on the strength of binding. Upon addition of guests (dissolved in CHCl_3_ containing 0.8% DMSO) the CHCl_3_ solution of **1** showed weak excimer only in presence of an excess concentration of guest acids. In this aspect, the change in fluorescence intensity of **1** in the presence of an excess concentration of citric and D-(−)-tartaric acids, dissolved in CHCl_3_ containing 0.8% DMSO, is represented by Figure 10 and Figure 11, respectively.

**Figure 12:**
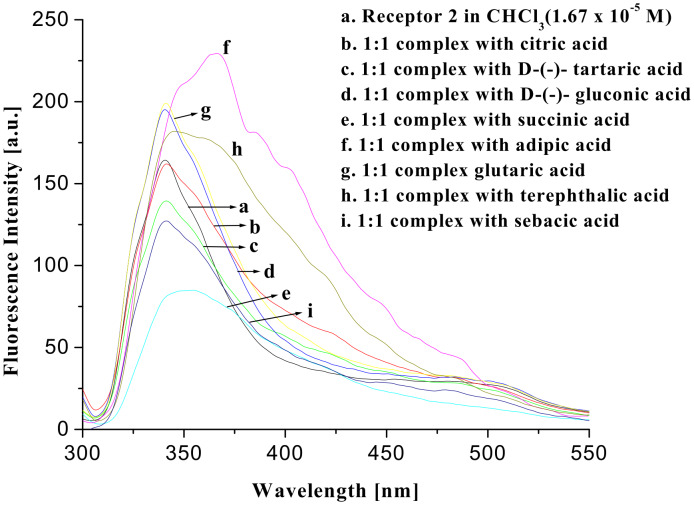
Fluorescence change of **2** in CHCl_3_ in the presence of carboxylic acids (λ_ex_ = 290 nm).

**Figure 13:**
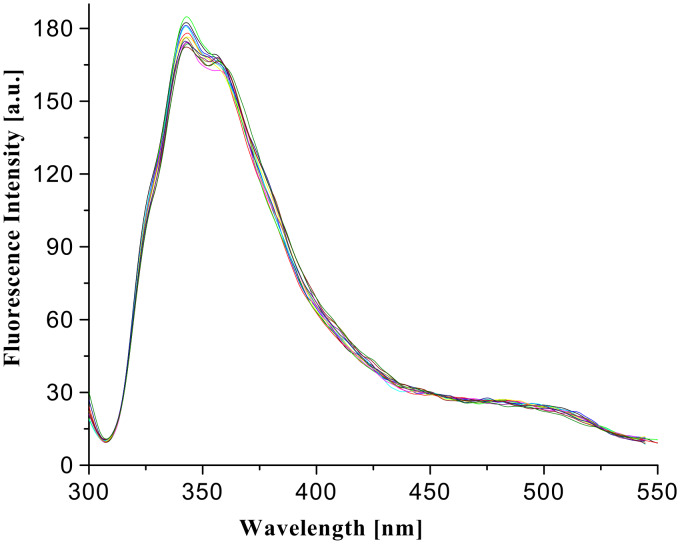
Fluorescence change of **2** in CHCl_3_ (*c *= 1.67 × 10^−5^ M) upon addition citric acid dissolved in CHCl_3_ containing 0.8% DMSO (λ_ex_ = 290 nm).

Interestingly, under similar conditions, the receptor **2** showed weak interaction with the same guests and did not produce a strong excimer upon complexation. As shown in Figure 12, the initially present less intense peak at higher wavelength (~ 500 nm) for weak excimer in **2** is marginally perturbed in the 1:1 complexes with the respective guests. Figure 13 indicates the change in fluorescence of **2** in CHCl_3_ upon gradual addition of citric acid, dissolved in CHCl_3_ containing 0.8% DMSO. It is of note that the change is insignificant compared to the case of the receptor **1** (see Figure 13). These observations prove the key role of quinoline in strong complexation of carboxylic acids, especially hydroxy acids.

#### ^1^H NMR study

**Figure 14:**
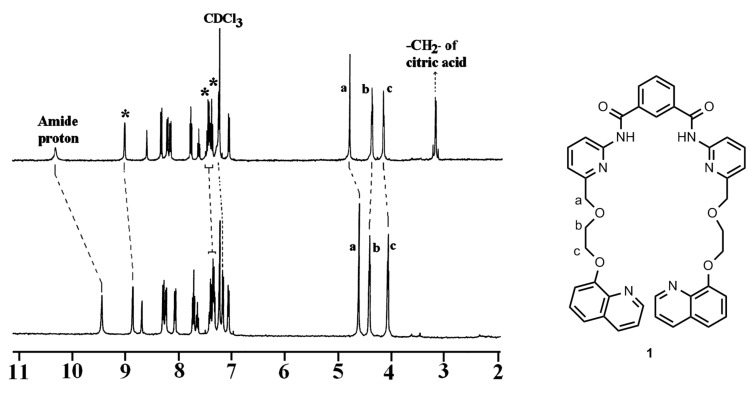
^1^H NMR (in CDCl_3_) spectra of receptor **1** (*c* = 3.57 × 10^−3^ M; bottom) and the 1:1 complex with citric acid (top); *indicates signals of quinoline rings.

To identify the possible hydrogen bonding sites and also to realize the conformational behavior of both **1** and **2**, ^1^H NMR spectra were recorded in CDCl_3_. To the receptor solutions in CDCl_3_, diacids were added in excess and the solutions were thoroughly sonicated for 10 min. Insoluble particles were removed by filtration and clear solutions were used to record the ^1^H NMR spectra. In all cases the complexes were of 1:1 stoichiometries, confirmed from the integration ratio of the receptor to the guest signals in ^1^H NMR. The receptor **1**, in CDCl_3_, showed a sharp peak at δ 9.45 ppm for the amide protons, which underwent a considerable downfield shift (Δδ = 0.21–1.08 ppm) upon addition of 1 molar equiv of the diacids studied, suggesting that aminopyridyl moieties serve as potential binding sites for carboxylic acids. The CH_2_ protons of the ethers, perfectly aligned into the cavity, also moved significantly downfield upon complexation. Among the three types of CH_2_ protons (a, b and c; see the structure **1**), types a and c moved more downfield (Δδ = 0.07–0.20 ppm) indicating a clear-cut case of H-bonding. This significant downfield shift of the CH_2_ protons (types a, c) of the ethers in the presence of the guest carboxylic acids (except terephthalic acid) in Table 1, led us to suggest that the lower rim of the receptor **1** is actively involved in complexation for which there is a substantial conformational change of the receptor **1**. As can be seen from Figure 14, a and c type protons of the ether chains undergo downfield shift upon complexation with citric acid. This subtle change via H-bonding presumably influences the quinoline groups to be close enough for formation of excimer. This is also evidenced by a change in the chemical shift values of the quinoline ring protons (Figure 14) during complexation with citric acid. The quinoline ring protons (marked with asterisks in Figure 14) suffer a downfield shift upon complexation and led us to presume a weak *edge* to *face* type π-stacking interaction between the pendant quinolines.

#### Theoretical calculations on receptors and selected complexes

In order to understand the flexible nature as well as modes of binding of the receptors **1** and **2** with the guest molecules studied in the present case, electronic structure calculations were carried out. Geometries of all compounds involved were subject to optimization at the AM1 level [21].

**Figure 15:**
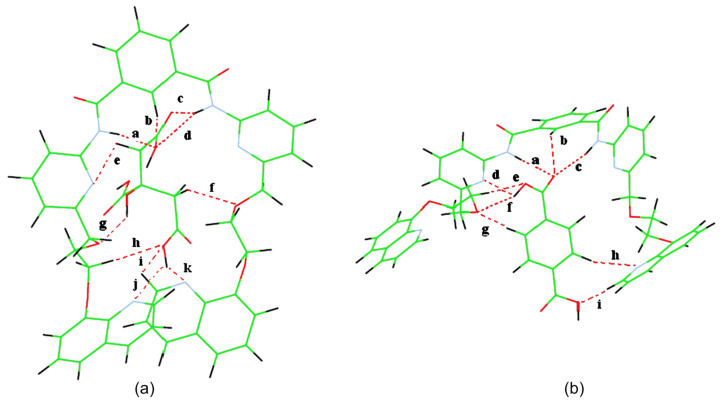
AM1 optimized geometries of the complexes of **1** with (a) citric acid, hydrogen bond distances: a = 2.16 Å, b = 2.57 Å, c = 2.16 Å, d = 2.36 Å, e = 2.96 Å, f = 2.38 Å, g = 2.26 Å, h = 2.32 Å, i = 2.47 Å, j = 2.65 Å, k = 2.94 Å and (b) terephthalic acid, hydrogen bond distances: a = 2.76 Å, b = 2.38 Å, c = 2.16 Å, d = 2.84 Å, e = 2.99 Å, f = 2.22 Å, g = 2.32 Å, h = 2.95 Å, i = 2.28 Å.

It is evident from the optimized geometry of the complex of **1** in Figure 15a that citric acid is strongly complexed in the cleft involving a large number of hydrogen bonding interactions. Both the amides as well as the isophthaloyl *peri* proton form hydrogen bonds with one of the carboxylic acid groups. The other terminal carboxylic acid forms bifurcated hydrogen bonds at the lower rim with the quinoline ring nitrogens and this is further stabilized by the adjacent three hydrogen bonds, formed from the participation of the ether oxygen of one arm, −OCH_2_− of another arm and one pendant quinoline ring hydrogen. The −CO_2_H group, attached to the carbon with an –OH group, remains uncomplexed. The −OH at the middle carbon forms a single hydrogen bond with the ether oxygen of one arm. Surprisingly, the methylene hydrogens of citric acids also form two hydrogen bonds. Such strong interaction brings the pendant quinolines close to exhibit a weak *edge* to *face* π-stacking interaction showing the shortest possible distance of 3.48 Å. The hydrogen bond distances are listed in Figure 15a.

In comparison, this weak π-stacking interaction between the quinolines is no longer found in the complex of **1** with terephthalic acid (Figure 15b). In the complex, the pendant quinolines are separated enough to form a cleft in which the carboxylic acid is complexed with the pyridine amide. The other carboxylic acid is singly bonded to one of the pendant quinolines via a ring hydrogen. One of the phenyl hydrogens of the guest terephthalic acid is bonded to the quinoline ring nitrogen. The hydrogen bond distances associated with this complex are listed in Figure 15b.

**Figure 16:**
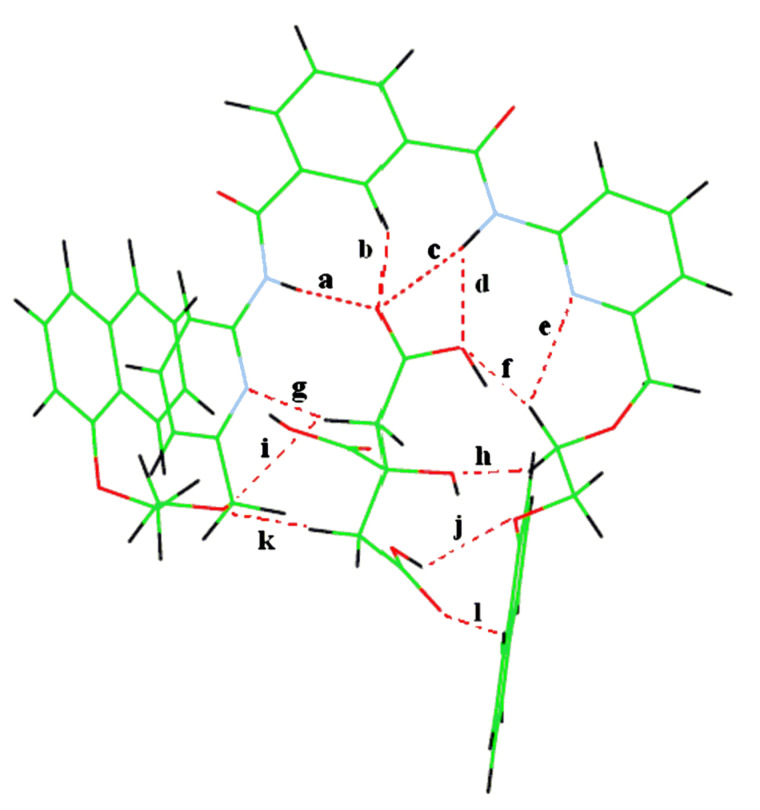
AM1 optimized geometry of the complex of **2** with citric acid, hydrogen bond distances: a = 2.12 Å, b = 2.39 Å, c = 2.19 Å, d = 2.36 Å, e = 2.66 Å, f = 2.92 Å, g = 2.69 Å, h = 2.32 Å, i = 2.93 Å, j = 2.25 Å, k = 2.25 Å, l = 2.38 Å.

We also did the same calculations on the complex of receptor **2** with citric acid. As can be seen from Figure 16, citric acid is complexed into the cleft with a number of hydrogen bonds and the pendant naphthalenes are separated by a large distance with no π-stacking interaction between them. This is in accordance with the experimental results, shown in the fluorescence experiment.

## Conclusion

We have discussed the synthesis and sensing behaviors of receptors **1** and **2** in the less polar solvent CHCl_3_. The conformationally flexible receptor **1** is able to distinguish hydroxy dicarboxylic acids from their non-hydroxy analogues and also aliphatic dicarboxylic acids from aromatic diacids by showing characteristic excimer emission, which is moderate and convenient for practical use. The findings have been explained by theoretical results. In the design **1**, quinoline ring nitrogen played a key role in the binding process and was established by doing the control experiments on the receptor **2**. The receptor **2** was found less effective than **1** in the binding and selection of the guest carboxylic acids.

## Experimental

*General details*. All reactions were carried out under a nitrogen atmosphere. Solvents were dried before use. Solvents for spectroscopic measurements were of spectroscopic or HPLC grade. THF was freshly distilled from sodium benzophenone ketyl. Et_3_N was distilled from KOH and stored over KOH pellets under nitrogen. Melting points were determined in open capillaries and are uncorrected. ^1^H NMR spectra were recorded on a Bruker 400 MHz spectrometer. UV-vis absorption and fluorescence spectra were recorded on a PerkinElmer Lambda 25 spectrophotometer and a PerkinElmer LS-50B spectrofluorimeter, respectively. FTIR spectra were obtained from a PerkinElmer L120-000A model.

### 2-(Quinolin-8-yloxy)ethanol (**3**)

To a mixture of 8-hydroxyquinoline (1 g, 6.80 mmol) and potassium carbonate (0.95 g, 6.80 mmol) in dry CH_3_CN, 2-chloroethanol (1.11 g, 13.7 mmol) in 5 mL CH_3_CN was added dropwise and the reaction mixture was refluxed for 24 h. The reaction mixture was concentrated under vacuum and 30 mL water was added to the mixture. The aqueous layer was extracted with 3 × 100 mL CHCl_3_ and the combined organic extracts were dried over anhydrous Na_2_SO_4_. After evaporation of the solvents under vacuum the crude product was purified by column chromatography using 25% ethyl acetate in petroleum ether as eluent to give a brownish crystalline product **3** (685 mg, yield 50%).

Melting point = 72 °C. ^1^H NMR (400 MHz, CDCl_3_, δ in ppm) 8.84 (1H, d, *J* = 2.40 Hz), 8.15 (1H, d, *J* = 8.00 Hz), 7.47–7.40 (3H, m), 7.11 (1H, d, *J* = 8.00 Hz), 5.54 (1H, br s, OH), 4.28 (2H, t, *J* = 4.00 Hz), 4.08 (2H, t, *J* = 4.00 Hz). ESI (positive ionisation) mass: 212.1 (M + Na)^+^, 190.1 (M + H)^+^. FTIR (ν cm^−1^, KBr) 3400, 3157, 2922, 1577, 1380, 1116.

### *N*-({6-[2-(Quinolin-8-yloxy)ethoxy]methyl}pyridin-2-yl)pivalamide (**4**)

To a solution of **3** (500 mg, 2.60 mmol) in dry THF, NaH (63 mg, 2.60 mmol) was added and stirred under a nitrogen atmosphere for 2 h. Then a solution of 2-(pivaloylamino)-6-bromomethylpyridine (0.72 g, 2.60 mmol) dissolved in 10 mL of THF was added and stirring was continued overnight. After completion of the reaction the reaction mixture was concentrated under vacuum and water (30 mL) was added to the reaction mixture. The aqueous layer was extracted with 3 × 100 mL CHCl_3_ and the combined organic extracts were dried over anhydrous Na_2_SO_4_. The solvent was evaporated and the crude product was purified by column chromatography using 20% ethyl acetate in petroleum ether as eluent to give compound **4** (702 mg, yield 70%) as a brownish gummy product.

^1^H NMR (400 MHz, CDCl_3_, δ in ppm) 8.93 (1H, dd, *J*_1_ = 1.60 Hz, *J*_2_ = 1.60 Hz), 8.29 (1H, s, -CONH-), 8.15–8.11 (2H, m), 7.65 (1H, t, *J* = 8.00 Hz), 7.46–7.38 (3H, m), 7.16 (1H, d, *J* = 8.00 Hz), 7.10 (1H, d, *J* = 7.60 Hz), 4.66 (2H, s), 4.46 (2H, t, *J* = 4.00 Hz), 4.11 (2H, t, *J* = 4.00 Hz), 1.32 (9H, s). ESI (positive ionisation) mass: 402.3 (M + Na)^+^, 380.2 (M + H)^+^. FTIR (ν cm^−1^, KBr) 3411, 1689, 1452, 1107.

### Receptor 1

To a solution of amide **4** (0.3 g, 0.79 mmol) in 20 mL ethanol, 10 mL of a 4 N KOH solution was added and the reaction mixture was refluxed for 12 h. After completion of the reaction (monitored by TLC) ethanol was evaporated and water (20 mL) was further added to the reaction mixture. The aqueous layer was extracted with 3 × 100 mL CHCl_3_ and the combined organic extracts were dried over anhydrous Na_2_SO_4_. The solvent was evaporated and the crude product was purified by column chromatography using 4% methanol in chloroform to give the corresponding amine **5** (175 mg, yield 75%). Without characterization, the amine **5** was directly used in the next step. Compound **5** (0.1 g, 0.30 mmol) was dissolved in dry CH_2_Cl_2_ and 0.15 mL of Et_3_N was added to it. Then this solution was added dropwise to a solution of isophthaloyl dichloride (0.034 g, 0.16 mmol) in dry CH_2_Cl_2_ and the reaction mixture was stirred overnight at room temperature. The solvent was evaporated and a saturated NaHCO_3_ solution (30 mL) was added to the reaction mixture. The aqueous layer was extracted with CHCl_3_ and the combined organic extracts were dried over anhydrous Na_2_SO_4_. The solvent was evaporated and the crude product was purified by column chromatography using ethyl acetate as eluent to give **1** (146 mg, yield 60%).

Melting point = 60 °C. ^1^H NMR (400 MHz, CDCl_3_, δ in ppm) 9.45 (2H, -CONH-), 8.87 (2H, d, *J* = 4.00 Hz), 8.70 (1H, s), 8.30 (2H, d, *J* = 8.24 Hz), 8.25 (2H, d, *J* = 7.64 Hz), 8.08 (2H, d, *J* = 8.28 Hz), 7.73 (2H, t, *J* = 8.00 Hz), 7.65 (1H, t, *J* = 7.76 Hz), 7.44–7.33 (6H, m), 7.18 (2H, d, *J* = 7.40 Hz), 7.07 (2H, d, *J* = 7.36 Hz), 4.64 (4H, s), 4.43 (4H, t, *J* = 4.00 Hz), 4.08 (4H, t, *J* = 4.00 Hz). ^13^C (100 MHz, CDCl_3_) 164.8, 155.9, 154.4, 151.4, 148.9, 139.8, 139.1, 136.1, 134.5, 131.5, 129.4, 129.3, 126.6, 125.8, 121.6, 119.8, 117.6, 113.2, 108.7, 73.3, 69.1, 67.8. ESI (positive ionisation): 743.3 (M + Na)^+^, 721.4 (M + H)^+^, 361.3, 296.2. FTIR (ν cm^−1^, KBr) 3385, 1677, 1455, 1318, 1105.

### 2-(Naphthalen-1-yloxy)ethanol (**6**)

To a mixture of 1-naphthol (1 g, 6.90 mmol) and potassium carbonate (0.96 g, 6.90 mmol) in dry CH_3_CN, 2-chloroethanol (1.12 g, 13.9 mmol) in CH_3_CN (5 mL) was added dropwise and the reaction mixture was refluxed for 60 h. After completion the reaction mixture was concentrated under vacuum and 30 mL water was added to the mixture. The aqueous layer was extracted with 3 × 100 mL CHCl_3_ and the combined organic extracts were dried over Na_2_SO_4_. After evaporation of the solvent the crude product was purified by column chromatography using 3% ethyl acetate in petroleum ether as eluent to give **6** as a deep brown gummy product (587 mg, yield 45%).

^1^H NMR (400 MHz, CDCl_3_, δ in ppm) 8.26 (1H, d, *J* = 7.20 Hz), 7.80 (1H, t, *J* = 6.80 Hz), 7.51–7.44 (3H, m), 7.36 (1H, t, *J* = 8.00 Hz), 6.83 (1H, d, *J* = 7.60 Hz), 4.27 (2H, t, *J* = 4.40 Hz), 4.10 (2H, br t), 2.10 (1H, br s, OH). ESI (positive ionisation): 211.1 (M + Na)^+^, 189.2 (M + H)^+^ . FTIR (ν cm^−1^, KBr) 2933, 1579, 2922, 1400, 1269.

### *N*-(6-{[2-(Naphthalen-1-yloxy)ethoxy]methyl}pyridin-2-yl)pivalamide (**7**)

To a solution of **6** (500 mg, 2.6 mmol) in dry THF, NaH (63 mg, 2.6 mmol) was added and the reaction mixture was stirred under nitrogen atmosphere for 2 h. Then the solution of 2-(pivaloylamino)-6-bromomethylpyridine (0.72 g, 2.6 mmol) dissolved in 10 mL of THF was added and stirring was continued overnight. After completion of the reaction, the reaction mixture was concentrated under vacuo and water (30 mL) was added to the reaction mixture. The aqueous layer was extracted with 3 × 100 mL CHCl_3_ and the combined organic extracts were dried over Na_2_SO_4_. The solvent was evaporated and the crude product was purified by column chromatography using 10% ethyl acetate in petroleum ether as eluent to give compound **7** as deep brown gummy product (744 mg, yield 74%).

^1^H NMR (400 MHz, CDCl_3_, δ in ppm) 8.28 (1H, d, *J* = 8.00 Hz), 8.19 (1H, s, -CONH-), 8.16 (1H, d, *J* = 8.40 Hz), 7.78 (1H, d, *J* = 8.00 Hz), 7.68 (1H, t, *J* = 8.00 Hz), 7.48–7.42 (3H, m), 7.35 (1H, t, *J* = 8.00 Hz), 7.22 (1H, d, *J* = 8.00 Hz), 6.82 (1H, d, *J* = 8.00 Hz) 4.69 (2H, s), 4.36 (2H, t, *J* = 4.80 Hz), 4.06 (2H, t, *J* = 4.80 Hz), 1.31 (9H, s). ESI (positive ionisation): 401.5 (M + Na)^+^, 379.2 (M + H)^+^. FTIR (ν cm^−1^, KBr) 3425, 2924, 1685, 1454, 1152.

### Receptor **2**

To a solution of amide **7** (0.4 g, 1.05 mmol) in ethanol (20 mL), 10 mL of a 4 N KOH solution was added and the reaction mixture was refluxed for 18 h. After completion of the reaction (monitored by TLC) ethanol was evaporated and water (20 mL) was added to the reaction mixture. The aqueous layer was extracted with 3 × 100 mL CHCl_3_ and the combined organic extracts were dried over anhydrous Na_2_SO_4_. The solvent was evaporated and the crude product was purified by column chromatography using 30% ethyl acetate in petroleum ether as eluent to give the corresponding amine **8** (243 mg, yield 78%). To a solution of amine **8** (0.15 g, 0.5 mmol) in dry CH_2_Cl_2_, Et_3_N (0.20 mL) was added and this solution was then added to a solution of isophthaloyl dichloride (0.05 g, 0.25 mmol) in dry CH_2_Cl_2_ with stirring. Stirring was continued overnight. After completion, solvent was evaporated and a saturated NaHCO_3_ solution (30 mL) was added to the reaction mixture. The aqueous layer was extracted with 3 × 100 mL CHCl_3_ and the combined organic extracts were dried over anhydrous Na_2_SO_4_. The solvent was evaporated and the crude product was purified by column chromatography using ethyl acetate as eluent to give **2** (190 mg, yield 52%).

Melting point = 58 °C. ^1^H NMR (400 MHz, CDCl_3_, δ in ppm) 8.94 (2H, s, -CONH-), 8.53 (1H, s), 8.25 (4H, d, *J* = 8.00 Hz), 8.15 (2H, d, *J* = 8.00 Hz), 7.78–7.74 (4H, m), 7.64 (1H, t, *J* = 8.00 Hz), 7.46–7.39 (6H, m), 7.33 (2H, t, *J* = 8.00 Hz), 7.27 (2H, d, *J* = 8.00 Hz), 6.80 (2H, d, *J* = 8.00 Hz), 4.70 (4H, s), 4.36 (4H, t, *J* = 4.40 Hz), 4.05 (4H, t, *J* = 4.00 Hz). ^13^C (100 MHz, CDCl_3_) 165.4, 156.3, 154.1, 151.5, 138.6, 134.2, 133.9, 131.6, 128.9, 127.2, 126.2, 126, 125.6, 125.3, 124.9, 121.7, 120.3, 117.3, 113.2, 104.6, 73.5, 69.1, 67.6. ESI (positive ionisation): 719.3 (M + H)^+^, 361. FTIR (ν cm^−1^, KBr) 3405, 1690, 1451, 1267, 1133.

### Methods for the determination of binding constant (*K*_a_) values by UV titration

#### a) Binding constant determination by dilution method

*General method:* The receptor was dissolved in 50 mL dry UV grade CHCl_3_. From this solution 25 mL was taken in a stoppered volumetric flask and to this the carboxylic acid guest was added and sonicated for 10 min. The mixture was filtered to remove any insoluble particle. Different solutions of varied compositions of receptor-carboxylic acid complex solution were prepared from this 25 mL stock solution of receptor-carboxylic acid complex by diluting with UV-grade CHCl_3_ maintaining the total volume to 10 mL. The different compositions (by volume) of receptor-carboxylic acid solution are: CHCl_3_ were 10:0, 7:3, 5:5, 3:7, 2:8, 1:9. UV-vis spectra were recorded for the receptor itself, the receptor-carboxylic acid complex solution and the different solutions of varied compositions of receptor-carboxylic acid solution. From the spectral data the binding constants were calculated for the carboxylic acids.

Working formula: d/A_c_ = (1/*K*_a_ ε_c_ l)^1/2^. 1/(A_c_)^1/2^ + 1/ε_c_l

Where d, A_c_, ε_c_ refer to the concentration, absorbance, molar extinction coefficient terms for the receptor-carboxylic acid complex.

The change in absorbance with different concentrations of the complex showed almost a linear dependence. This indicated the 1:1 stoichiometry of the complex.

#### b) Binding constant determination by continuous variation method

*General method*: Binding constants were determined by UV-vis titration methods. Initially the receptor was dissolved in dry UV grade chloroform and taken in the cuvette. Then carboxylic acid guests, dissolved in dry CHCl_3_ containing 0.8% DMSO, were individually added in different amounts to the receptor solution. The corresponding absorbance values during titration were noted and used for the determination of binding constant values. Binding constants were determined by using the expression A_0_/A − A_0_ = [ε_M_/(ε_M_ − ε_C_)](*K*_a_^−1^C_g_^−1^ + 1), where ε_M_ and ε_C_ are molar extinction coefficient for receptor and the hydrogen- bonding complex, respectively at selected wavelength, A_0_ denotes the absorbance of the free receptor at the specific wavelength and C_g_ is the concentration of the carboxylic acid guest. The measured absorbance A_0_/A − A_0_ as a function of the inverse of the carboxylic acid guest concentration fits a linear relationship, indicating a 1:1 stoichiometry of the receptor-carboxylic acid complex. The ratio of the intercept to the slope was used to determine the binding constant *K*_a_.
